# Investigation on a focus of human trichinellosis revealed by an atypical clinical case after wild-boar (*Sus scrofa*) pork consumption in northern Italy

**DOI:** 10.1051/parasite/2011181085

**Published:** 2011-02-15

**Authors:** F. Romano, A. Motta, M. Melino, M. Negro, G. Gavotto, L. Decastelli, E. Careddu, C. Bianchi, D.M. Bianchi, E. Pozio

**Affiliations:** 1 SIAN ASL CN1 Mondovì Italy; 2 SVET ASL CN1 Mondovì Italy; 3 Family physician Roccaforte, Mondovì Italy; 4 Istituto Zooprofilattico Sperimentale of Piemonte Liguria and Valle d’Aosta, Turin Italy; 5 National Reference Laboratory for Trichinella, Istituto Superiore di Sanità Rome Italy

**Keywords:** trichinellosis, wild boar, epidemiology, *Trichinella britovi*, humans, Italy, trichinellose, sanglier, épidémiologie, *Trichinella britovi*, homme, Italie

## Abstract

Trichinellosis is one of the most serious foodborne parasitic zoonoses in Europe. Wild carnivorous and omnivorous hosts are the main reservoirs of *Trichinella* spp. nematodes in nature. In the winter of 2008-2009, an atypical clinical case of trichinellosis occurred for the consumption of pork from a wild boar (*Sus scrofa*) hunted in southwestern Alps in Italy. The symptomatic individual showed delayed development of oedemas in the lower limbs and eosinophilia, which appeared three months after infection. Muscle samples harboured 3.8 larvae/g, which were identified as *Trichinella britovi*. During the epidemiological investigation, anti-*Trichinella* IgG were detected in five hunters.

## Abstract (fr)

La trichinellose est l’une des plus importante zoonose parasitaire transmise par les aliments en Europe. Les animaux sauvages et les hôtes omnivores sont les importants réservoirs des nématodes du genre *Trichinella* spp. Durant l’hiver 2008-2009, un cas de trichinellose a été causé par la consommation de viande d’un sanglier (*Sus scrofa*) chassé dans le sud-ouest des Alpes en Italie. La syptomatologie de la personne infectée a été tardive, avec un oedème des membres inferieures et une hyperéosinophilie qui sont apparus trois mois après la consommation. Les échantillons des muscles examinés hébergeaient 3,8 larves/g, identifiées comme *Trichinella britovi*. Au cours de l’étude épidémiologique, des IgG anti- *Trichinella* ont été détectées chez cinq chasseurs asymptomatiques.

Trichinellosis is a zoonotic disease caused by nematode worms of the genus *Trichinella* (Gottstein *et al*., 2009). It is a worldwide parasitosis caused by the consumption of raw or semi-raw meat infected with larvae (Pozio *et al*., 2007). In the European Union, in the past 30 years trichinellosis due to the consumption of local meat products, mainly from backyard pigs and game meat, has been documented in Bulgaria, Estonia, France, Germany, Greece, Hungary, Italy, Latvia, Lithuania, Poland, Romania, Slovakia and Spain, though the reported prevalence varies (Pozio *et al*., 2007). In Italy, since World War II most cases of trichinellosis (73.3 %) have been caused by horse meat imported from third countries, whereas the consumption of pork or pork-derived products from backyard or free-ranging pigs and from hunted wild boars have only accounted for 11 % and 6.5 % of cases, respectively. In Italy, the most common etiological agent of *Trichinella* infection in humans and animals is *Trichinella britovi*, which is maintained in nature by a sylvatic cycle in which the red fox (*Vulpes vulpes*) is the main reservoir (Pozio *et al*., 2009). The objective of the present work was to describe an atypical clinical case of trichinellosis which occurred in 2008 due to the consumption of raw pork from a wild boar (*Sus scrofa*), and the epidemiological investigation in the Piedmont Region of Italy.

## The Index Case

In December 2008, a 51-year-old woman was admitted to the Department of Internal Medicine of the *S. Croce e Carle* Hospital in Cuneo (Piedmont Region, northern Italy) for oedemas, myalgia, muscle weakness and functional impotence in the lower limbs. One month before hospitalization, she had had a single event of hyperthermia (with a body temperature of up to 38.5 °C) and diarrhoea for two days. Kidney and hart functions were normal. Laboratory data showed increased transaminases and muscle enzymes (CK and LDH), but no eosinophila was observed. The level of blood albumin was 17.20 g/l. No anti-DNA antibodies were detected. The anamnesis showed that the patient, before to acquire trichinellosis, was already affected by a pancreatic failure due to a partial pancreatectomy carried out in 1991, and her general conditions were not good due to hypoalimentation and hepatic steatosis. After a few days of hospitalization and without any pharmacological treatment, there was a reduction in the signs and symptoms in the lower limbs, and the patient was discharged without a diagnosis. Two months later, she was hospitalised again for oedemas in the lower limbs and eosinophilia (4.245 × 103 μl). A muscle biopsy resulted positive for the presence of coiled nematode larvae surrounded by a collagen capsule. The woman was treated with albendazole (400 mg × two times per day for 7 days) and prednisone (25 mg per day for 7 days), and she promptly recovered.

## Epidemiological Investigation

The epidemiological investigation revealed that the woman’s husband was a hunter and that game meat (mainly pork from wild boars) had been frequently consumed at home. The woman reported that although she frequently prepared and froze the game meat, she was a vegetarian and did not consume the meat. However, she admitted to have tested the minced meat during the preparation of pork and pork derived products (sausages and salami) from wild boars hunted by her husband.

Blood samples were collected from 36 persons (hunters and their friends and relatives) who could have consumed pork or pork products from hunted wild boars. Serum samples were tested using an ELISA, and the positive samples were confirmed with Western blot, according to previous published protocols (Gomez Morales *et al.*, 2008). Five serum samples (13.9 %) from the patient’s husband who is a wild boar hunter, and from four other hunters of the same hunting team, were confirmed to be positive. None of the five serologically positive persons reported that they had had clinical signs or symptoms suggestive of trichinellosis in the previous six months.

The woman and her family as well as the serologically positive hunters lived in Mondovì, a small town of about 25,000 inhabitants in the Province of Cuneo (Piedmont Region). The local veterinarian and personnel of SIAN services visited the woman’s home and collected pork samples from three different wild boars stored in the freezer. These animals had been hunted in November and December 2008 in the game reserve of Mondolè, a mountain area of the southwestern Alps (latitude 44°13’22’’N, longitude 7°45’21’’E). The veterinarian and SIAN services contacted the six hunters who had participated in the hunting expedition the day that the infected wild boar had been caught and alerted them as to the risk of acquiring trichinellosis; they also asked the hunters to handover all of the wild boar meat in their freezers and the products (*e.g*., salami and sausages) made with the wild boar meat.

Aliquots of muscle tissue from the three wild boars collected in the patient’s home, were tested for *Trichinella* in accordance with the Commission Regulation (EC) 2075/2005. Muscle samples from a wild boar hunted in November and frozen on November 23^rd^ tested positive for the presence of *Trichinella* larvae (3.8 per gram). The larvae were identified as *T. britovi* by a multiplex PCR test (Pozio & Rosa, 2003).

Twenty three aliquots of frozen muscle samples and 16 salamis made with pork from wild boars hunted on the same day as the *Trichinella*-positive wild boar were collected at the homes of the six hunters and tested by artificial digestion, as reported above. Three aliquots of muscle samples and three salamis collected in the houses of two hunters tested positive for *Trichinella* larvae. Unfortunately, it was not possible to establish whether the positive samples originated from only one wild boar or from more than one. None of the larvae collected from the frozen muscles or the salamis showed any movement after artificial digestion, suggesting that they had died about six months after freezing or salami preparation. From September to December 2009, some local hunters requested that the wild boars hunted in the Mondolè game reserve during this period be tested for *Trichinella*; most animals (n = 85) were tested, yet none of them resulted positive.

## Discussion

This is the sixth documented focus of trichinellosis for the consumption of pork from hunted wild boars in Italy. The first focus, which involved six persons, occurred in 1978 in the Oliveto Lucano municipality (Basilicata Region, southern Italy) (Lo Nigro *et al*., 1978). The second focus, which involved 48 persons, occurred in 1988 in the Polino municipality (Umbria Region, central Italy) (Frongillo *et al*., 1992). Three outbreaks, which involved a total of 35 persons, occurred in 1995, 1996 and 2002 in the Castel di Sangro (two outbreaks) and Popoli (one outbreak) municipalities (Abruzzi Region, central Italy) (Pozio E., unpublished data).

The prevalence of *T. britovi* in the wild boar population of Italy is low. From 1988 to 2009, *T. britovi* was detected in only 14 wild boars, hunted in the Regions of Abruzzi (n = 5), Emilia Romagna (n = 2), Liguria (n = 2), Marche (n = 1), Umbria (n = 2) and Valle d’Aosta (n = 2) (International *Trichinella* Reference Centre, www.iss.it/site/Trichinella/index.asp). One of the main reasons for this low prevalence could be the low susceptibility of wild boars to *T. britovi*. In fact, experimental infections have shown that no larva is detectable in muscles of *T. britovi*-infected swine 6-8 months post-infection (Nöckler K., personal communication). *T. britovi* larvae can survive in frozen meat from a wild boar up to three weeks at -20 °C (Pozio *et al*., 1992).

The clinical picture observed for the index case is uncommon. The lack of eosinophilia one month after infection and the fluctuating oedema and myalgia of the lower limbs up to four months after infection have never been documented in *Trichinella*-infected individuals (Dupouy-Camet & Bruschi, 2007). This atypical clinical pattern of trichinellosis could be the results of four concomitant causes: 1. the pancreatic failure, hypoalimentation and hepatic steatosis; 2. the low amount of larvae per gram in pork; 3. the low amount of ingested larvae during pork preparation; and 4. the low pathogenicity of *T. britovi*.

The detection of anti-*Trichinella* IgG in five asymptomatic individuals is consistent with previous reports that *T. britovi* is less pathogenic to humans than other *Trichinella* species, because of the low number of larvae produced by each female and their lower immunogenicity, but also can suggest that these five asymptomatic people, all of whom were hunters, ingested low doses of *Trichinella* larvae more times during their life. It is well known that anti-*Trichinella* IgG can persist in humans for more than 10 years (Harms *et al*., 1993) if the larvae are not killed by an appropriate treatment. Alive *Trichinella* larvae have been detected in muscle biopsies 39 years post infection (Fröscher *et al*., 1988). Furthermore, in persons with mild infection, it could be difficult to distinguish trichinellosis from other infectious diseases, such as influenza. In fact, most of the *Trichinella* infections caused by the consumption of pork occur in the cold season, when other infectious diseases such as influenza, are quite common, and there are no pathognomonic signs and symptoms of trichinellosis. Unfortunately, no information was available on the amount of pork consumed by each of the serologically positive individuals, which could be useful for roughly estimating the number of larvae able to induce an asymptomatic infection.
